# Contrasting growth, physiological and gene expression responses of *Clematis crassifolia* and *Clematis cadmia* to different irradiance conditions

**DOI:** 10.1038/s41598-019-54428-z

**Published:** 2019-11-28

**Authors:** Xiaohua Ma, Renjuan Qian, Xule Zhang, Qingdi Hu, Hongjian Liu, Jian Zheng

**Affiliations:** Institute of Subtropical Crops of Zhejiang Province, Wenzhou, Zhejiang 325005 China

**Keywords:** Light responses, Abiotic

## Abstract

*Clematis crassifolia* and *Clematis cadmia* Buch.-Ham. ex Hook.f. & Thomson are herbaceous vine plants native to China. *C. crassifolia* is distributed in shaded areas, while *C. cadmia* mostly grows in bright, sunny conditions in mountainous and hilly landscapes. To understand the potential mechanisms involved in the irradiance responses of *C. crassifolia* and *C. cadmia*, we conducted a pot experiment under three irradiance treatments with natural irradiation and two different levels of shading. Various growth, photosynthetic, oxidative and antioxidative parameters and the relative expression of irradiance-related genes were examined. In total, 15 unigenes were selected for the analysis of gene expression. The exposure of *C. crassifolia* to high irradiance resulted in growth inhibition coupled with increased levels of chlorophyll, increased catalase, peroxidase, and superoxide dismutase activity and increased expression of c144262_g2, c138393_g1 and c131300_g2. In contrast, under high irradiance conditions, *C. cadmia* showed an increase in growth and soluble protein content accompanied by a decrease in the expression of c144262_g2, c133872_g1, and c142530_g1, suggesting their role in the acclimation of *C. cadmia* to a high-irradiance environment. The 15 unigenes were differentially expressed in *C. crassifolia* and *C. cadmia* under different irradiance conditions. Thus, our study revealed that there are essential differences in the irradiance adaptations of *C. crassifolia* and *C. cadmia* due to the differential physiological and molecular mechanisms underlying their irradiance responses, which result from their long-term evolution in contrasting habitats.

## Introduction

*Clematis* (Flora of China 6)^1^ species include diverse groups of perennial woody and herbaceous vine plants that belong to the Ranunculaceae family. *Clematis* is a large genus within the dicotyledons, and approximately 355 species are known worldwide. China is rich in *Clematis* diversity, with 147 species listed in the Flora of China^2,3^ and approximately 110 species widely distributed in Southwest China^4^.

Due to long-term evolution under diverse habitats that dictate resource availability and successional specificitie*s*^5^, different species of *Clematis* have adapted to survive in their particular ecological niches. Thus, the entire *Clematis* genus contains many species that embody extremely different biological characteristics and growth habits^6,7^.

*Clematis* species are widespread in the Northern Hemisphere, and most of them have been used extensively in traditional medicines around the world^8,9^. *Clematis* has also been extensively used for ornamental purposes in recent years^10^. A wide range of color and flower shapes are found across many *Clematis* varieties and species, and therefore, it has been called “the queen of vines”^11^. For instance, sweet autumn *Clematis*(*C. maximowicziana*, *C. paniculata)* is a vigorous species popular for its masses of fragrant white flowers. Large-flowered *Clematis*(*Clematis patens)* is widely used and well known for its large flowers and rich colors^10^.

*C. crassifolia* is an evergreen species that is extensively used in urban landscapes and home gardens. It can grow vigorously in cold winter conditions. In southern China, *C. crassifolia* flowers in winter when the temperature is between 4 °C and 10 °C and the soil is cool and moist, which makes it stand out from many other cultivars and species of *Clematis*. *C. cadmia* is loved by gardening professionals for its copious white flowers and excellent tolerance to adverse conditions and could also be exploited as a source of stress-resistant genes for *Clematis* breeding.

It is well known that different plant species can endure different irradiance stresses. *C. cadmia* mostly prefer high-light environments in daily production. *C. crassifolia* is a special *Clematis* species and usually has a longer and slower growth rate than other *Clematis* species. This suggests that different light requirements could exist between *C. cadmia* and *C. crassifolia*. Thus, it is necessary to research the physiological and biochemical differences between *C. cadmia* and *C. crassifolia* under different irradiance environments.

Suboptimal irradiance can be one of the major limiting factors restricting the growth and development of plants^12^. Plants are under illumination stress when the available light is either in excess or deficient. High irradiance stress can exacerbate ROS production, which overwhelms the ROS scavenging system and generates various secondary messengers, resulting in photoinhibition, photoinactivation and photodamage in plant cells^13,14^. However, plants grown under insufficient irradiance levels often suffer reduced ribulose-1,5-bisphosphate carboxylase oxygenase (Rubisco) activity and low CO_2_ assimilation rates, thereby leading to reduced photosynthetic productivity^15^.

The consequences of irradiance stress on the whole plant are quite complex and relate to physiological and metabolic functions and molecular responses. Mittler^16^ found that the generation of ROS in plants is usually low under normal growth conditions, whereas enhanced production of ROS occurs under high or low irradiance stress conditions. Increased ROS is known to cause damage to the photosynthetic apparatus as well as to other metabolic systems.

Moreover, in recent years, many genes involved in plant growth and physiological responses to light have been investigated, and stimulus-specific changes in gene expression are often observed when plants acclimate to adverse light environments^17,18^. Gau *et al*.^19^ isolated a single-copy gene (*psbY*) encoding PsbY-A1 and PsbY-A2, which are present in PS II core complexes and in reaction center complexes, from spinach. They observed that PsbY was involved in photosynthesis and the absorption of light energy in plants, and thus, its expression was significantly affected by light intensity. Evidence shows that irradiance stress conditions induce a primed state in plants that enhances gene expression, including the expression of defensive genes that protect plants against the adverse effects of high or low irradiance, genes that encode signaling proteins related to induced systemic resistance (ISR) pathways and a sequence of genes that are involved in the process of photosynthesis in plants^20,21^. However, most studies on irradiance have focused on physiological activities in plants, whereas less attention has been paid to the response to irradiance in gene expression.

Previous studies on *Clematis* often focused on the chemical constituents, pharmacology, toxicology and clinical studies of *Clematis* species^4,22–24^. There are few published studies on growth, physiology and acclimatory variation at the molecular level in *Clematis* species under different environmental conditions. Our preliminary experiments on the survival and early growth of the genus *Clematis* indicated that *Clematis* species growth may be strongly affected by irradiance. The objectives of our study are (i) to investigate the effects of irradiance on photosynthesis, growth and development in *C. crassifolia* and *C. cadmia*, (ii) to elucidate how defensive enzymatic activity and gene expression respond to different irradiance levels, and (iii) to determine the irradiance levels that optimize the growth of *C. crassifolia* and *C. cadmia*. We focused on various physiological, biochemical and molecular responses in both species under different irradiance levels, which is expected to provide a theoretical foundation for the development of beneficial management practices for *C. crassifolia* and *C. cadmia*.

## Materials and Methods

### Plant materials and growth conditions

**Figure 1:**
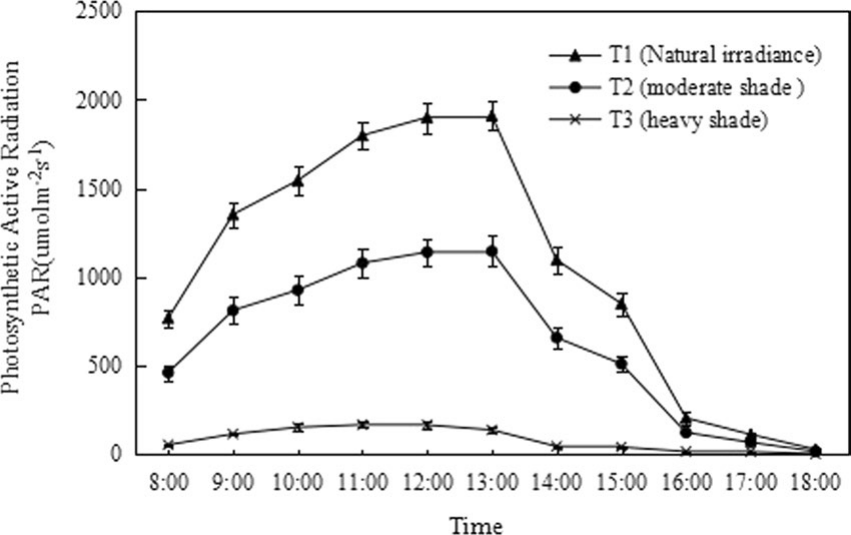
Diurnal variation of photosynthetically active radiation (PAR) under natural irradiance (T1), moderate shade (T2); and heavy shade (T3) conditions. The values are means ± SE of the PAR over the growing period.

A pot experiment was established using shading nets of different thicknesses in a room at the Zhejiang Institute of Subtropical Crops, Zhejiang Province, China (N28°23′, E120°72′). Seeds of one-year-old healthy and uniform *Clematis* seedlings (*C. crassifolia* and *C. cadmia*, 8- to 10-leaf age) were individually transferred to plastic pots (16.5 cm inner diameter, 18 cm height, with holes in the bottom) filled with a substrate mixture of perlite:peat:black soil (2:5:3, v/v/v, 45 kg m^−3^ of organic manure). After six months, the potted plants were grown under a temperature range of 20–32 °C and three different irradiance levels, (i) natural irradiance (T1), (ii) moderate shade (T2), and (ii) heavy shade (T3) (Fig. 1). The different irradiances were established using different layers of commercial black shading net (resembling square tents, commonly used in agriculture), and the irradiance was measured using a digital lux meter (TES-1339R, Taiwan) for a week and averaged. Planted pots were rotated daily to minimize the effect of the environment and irrigated daily to keep the plants well-watered (the water level was kept at 75% of the field capacity of the soil). After three months of growth, leaves were selected, and a completely randomized design with five replications per treatment was established. Each irradiance treatment included the two species of *Clematis*, *C. crassifolia* (C1) and *C. Cadmia* (C2). The treatments were as follows: T1C1 (*C. crassifolia* grown under natural irradiance conditions); T2C1 (*C. crassifolia* grown under moderate shade irradiance conditions); T3C1 (*C. crassifolia* grown under heavy shade conditions); T1C2 (*C. cadmia* grown under natural irradiance conditions); T2C2 (*C. cadmia* grown under moderate shade irradiance conditions); and T3C2 (*C. cadmia* grown under heavy shade conditions). Thus, the experiment was set up in a split-plot design, in which irradiance levels constituted the main plots and *Clematis* species were the subplots, and contained five replicates with twenty-five pots each.

### Leaf growth analysis

After three months of the differential irradiance treatments, one intact plant from each replication of each treatment was selected to observe and record the growth conditions of the whole plant. The leaf samples of *Clematis* from all treatments were collected for growth analysis. The leaf mass was weighed by electronic scale, and the leaf area was measured by an LA meter (LI-300, Li-Cor, Lincoln, NE, USA). The leaves were killed by keeping them at 105 °C for 15 min and then dried in an oven at 80 °C until they reached a constant dry weight. Specific leaf weight (SLM) was calculated by leaf dry mass divided by the leaf area for each plant^25^.

### Chlorophyll content analysis

Chlorophyll content was measured as described by Lichtenthaler with a spectrophotometer (Shimadzu UV-2550, Kyoto, Japan) and expressed as mg g^−1^ fresh weight (FW)^26^.

### Gas exchange measurements

Healthy and fully developed leaves from each treatment were chosen for photosynthetic parameter measurements with a LI-6400 XT portable photosynthesis system (LI-COR Inc., Lincoln, NE, USA) equipped with a 6400-18 RGB LED light source. The measurements were carried out on sunny days from 9:30 to 11:00 am at an air concentration of 21% O_2_, 1200 µmol m^−2^s^−1^ photosynthetically active radiation (PAR) white light, 400 µmol of CO_2_ mol^−1^ of dry air, 55% relative humidity and a temperature range of 28–32 °C^27^.

### Cellular membrane damage analysis

Cell membrane stability and integrity were expressed as the relative electrical conductivity (REC) and were measured as previously described^28^. The membrane peroxidation was expressed as the malondialdehyde (MDA) content and was measured by the method of Hodges *et al*.^29^.

### Peroxide (H_2_O_2_) content and superoxide anion (O^−2^) production rate analysis

The H_2_O_2_ content was determined with the method described by Patterson *et al*.^30^ and expressed as mM mg^−1^ FW. The superoxide anion (O^.−2^) production rate was determined using the method of Wang and Luo^31^ and expressed as µM min^−1^ mg^−1^ FW.

### Antioxidant enzyme activity analysis

Leaf samples (1.0 g FW) were ground in a mortar containing 8.0 ml of grinding media consisting of 1% polyethylene pyrrole (PVP) and 50 mM phosphate buffer solution (pH 7.4) at 4 °C. Following centrifugation at 10,000 rpm for 15 min at 4 °C, the supernatants were collected to obtain crude enzymes. Total superoxide dismutase (SOD) activity was determined spectrophotometrically at 520 nm and expressed as U g^−1^ FW^32^. Catalase (CAT) activity was determined by monitoring the disappearance of H_2_O_2_, and specific CAT activity was expressed as U g^−1^ FW min^−1^ ^33^. The peroxidase (POD) activity was measured with the method described by Thomas *et al*., and specific POD activity was expressed as U g^−1^ FW min^−1^ ^34^.

### Soluble protein content analysis

The soluble protein content was measured with Coomassie brilliant blue staining according to the method described by Bradford and was expressed as mg g^−1^ FW^35^.

### Related gene expression analysis

We screened various genes related to the irradiance stress response through transcriptome sequencing to analyze gene expression (Table 1). Primer Premier 5 (www.Premierbiosoft.com/primerdesign) was used for designing primers. Table 2 shows the primer information for amplification of the analyzed genes. Total RNA was extracted from frozen and pulverized Clematis leaves using an RNeasy column (Qiagen USA, California, CA), and RNA samples for each replicate were pooled to obtain a single RNA sample for cDNA and cRNA probe preparation and expression profiling. qRT-PCR experiments were conducted as described by Gao *et al*. using Real Master Mix (SYBR Green) (Roche Applied Science, Mannheim, Germany)^36^.

**Table 1:** The unigene annotations in the KEGG pathway database.

Gene code	Gene id	Description
c136757_g1	gi|225467859	chlorophyllase-1
c145729_g1	gi|720029945	chloroplastic-like
c144230_g2	gi|944542068	photosystem II protein D (chloroplast)
c144262_g2	gi|224110818	Photosystem II core complex proteins psbY
c136259_g1	gi|720003112	abscisic acid 8&apos;-hydroxylase 2-like
c135142_g1	gi|225439530	abscisic acid receptor PYL4
c144435_g2	gi|659130926	superoxide dismutase [Cu-Zn], chloroplastic
c142929_g5	gi|719994589	auxin-induced protein AUX22
c139448_g1	gi|720071799	auxin-responsive protein IAA4-like
c131300_g2	gi|643707025	abscisic acid receptor PYL4
c142530_g1	gi|734405933	Indole-3-acetic acid-induced protein
c133872_g1	gi|720061581	abscisic acid receptor PYR1
c144154_g3	gi|747049105	protein TIFY 7
c151338_g2	gi12620328	tyrosine/dopa decarboxylase
c138393_g1	gi|723802077	transcription factor TGA2

**Table 2:** Primer information for the quantification of gene expression by qRT-PCR.

Genes code	Primer	Sequences	Annealing temp (°C)	Gene length
actin	actin-F	AACCCTGAGGAGATTCCA	60	162
	actin-R	CACCACCCTTCAAGTGAGCAG		
c136757_g1	c136757_g1_1F	CGGCTATGTAATCGTTGCT	62	298
	c136757_g1_1R	TAATGCGAGAGAGAATGCC		
c145729_g1	c145729_g1_1F	CCTTCAACATCTCTACCTCCA	64	267
	c145729_g1_1R	TAACCTTCGTCCCACTCCT		
c144230_g2	c144230_g2_1F	CCAAGATTTTACCATGACTGC	62	119
	c144230_g2_1R	AAACACCGAACCATCCAA		
c144262_g2	c144262_g2_1F	CTCCAAACCAACTCATCTCC	63	161
	c144262_g2_1R	ACTCAAAGCGGCAAATACA		
c131300_g2	c131300_g2_1F	CTGAGGAAAGTTGAGGTGGT	64	142
	c131300_g2_1R	TGGTCACGGAACGGTAGT		
c142530_g1	c142530_g1_1F	GACTCGGTGGTTCCAAAA	62	133
	c142530_g1_1R	CAAATTCTTCCTCGGCTCT		
c133872_g1	c133872_g1_1F	CAAGCCCCAAACCTACAA	62	194
	c133872_g1_1R	GATGCTCTCCGCCAATAA		
c144154_g3	c144154_g3_1F	CAAAGTCTGCGGTAGCATC	64	280
	c144154_g3_1R	ACAGGTGGTTCGGTATTGTT		
c151338_g2	c151338_g2_1F	CACAACAGGCACAACATCA	63	272
	c151338_g2_1R	GGCAACAACAATCCAAAGTAG		
c138393_g1	c138393_g1_1F	CAGATGGCTCTTGCTCTCA	63	265
	c138393_g1_1R	AATGACTGTGCTGAACGATTT		
c136259_g1	c136259_g1_1F	ATCTTCCTTTCTGCCTGCT	63	100
	c136259_g1_1R	AGTGTTGATGGTAGCGTTTTC		
c135142_g1	c135142_g1_1F	TTATTCGGAGATGGCAAAGT	61	217
	c135142_g1_1R	CAACATACGATTCAACCACAA		
c144435_g2	c144435_g2_1F	TCTACAGGGGCACACTTCA	65	280
	c144435_g2_1R	TCAGTCCAACAACACCACA		
c142929_g5	c142929_g5_1F	CCTGAAGAATCACAAAGGGT	62	152
	c142929_g5_1R	CTCCAACAAGCATCCAGTC		
c139448_g1	c139448_g1_1F	AAAACAGCATCCAAGCGA	62	252
	c139448_g1_1R	CATCTCCAACCAACATCCA		

### Data analysis

Statistical analysis was conducted using a two-factor analysis of variance with SPSS software version 16.0 (SPSS, Chicago, IL, U.S.). Tukey’s multiple range test was applied to detect differences between the means. The data shown are the mean ± standard deviation (SD).

## Results

### Plant growth and development

**Figure 2:**
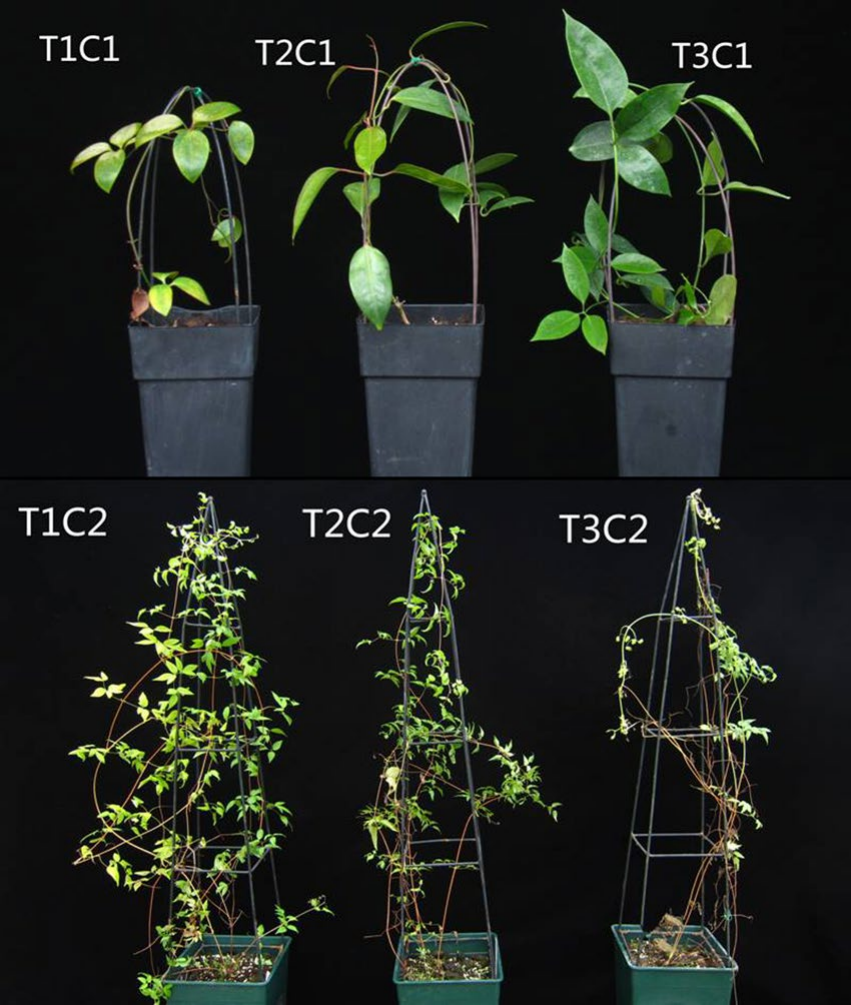
Growth phenotypes of in *C. cadmia* plants and *C. crassifolia* plants under three levels of irradiance levels including natural irradiance (T1), moderate shade (T2); and heavy shade (T3) condition.

The *C. crassifolia* and *C. cadmia* grown under different irradiances for three months are shown in Fig. 2. Irradiance had different effects on the leaf growth of *C. crassifolia* and *C. cadmia*. Additionally, irradiance levels significantly affected the leaf growth of *Clematis* (p < 0.05) (Table 3). After three months of irradiance treatment, the total leaf fresh weight per seedling of *C. crassifolia* plants increased by 8.5% (p < 0.05) and 21.78% (p < 0.05), respectively, under T2 and T3 light treatments compared with that of plants subjected to T1 irradiance treatments. Conversely, the leaf fresh weight of *C. cadmia* decreased by 25.82% (p < 0.05) and 52.23% (p < 0.05) under T2 and T3 irradiances, respectively, compared with that under T1 treatments. However, both *C. crassifolia* and *C. cadmia* exhibited a significant decrease (p < 0.05) in SLW with decreasing irradiance.

**Table 3:** Leaf fresh weigh and specific leaf weight (SLW) of *C. crassifolia* (C1) *and C. Cadmia* (C2) plants subjected to three different levels of Irradiance including natural irradiance (T1), moderate shade (T2); and heavy shade (T3).

Treatment	Specific leaf weight(g/cm^2^)	leaf fresh weight(g)
T1C1	2.71 ± 0.07Aa	13.76 ± 0.98Ac
T2C1	1.99 ± 0.08Ab	16.55 ± 1.62Ab
T3C1	1.48 ± 0.06Ac	19.49 ± 1.08Aa
T1C2	1.28 ± 0.02Ba	6.54 ± 0.94Ba
T2C2	0.73 ± 0.03Bb	4.82 ± 0.71Bb
T3C2	0.52 ± 0.03Bc	3.14 ± 0.52Bc

Different uppercase letters indicate significant difference between different *Clematis* species under same irradiance treatment at 0.05 levels; lowercase letters indicate significant difference within the same *Clematis* species under different irradiance treatment at 0.05. The values presented are the means ± SE.

### Photosynthetic pigments

**Figure 3:**
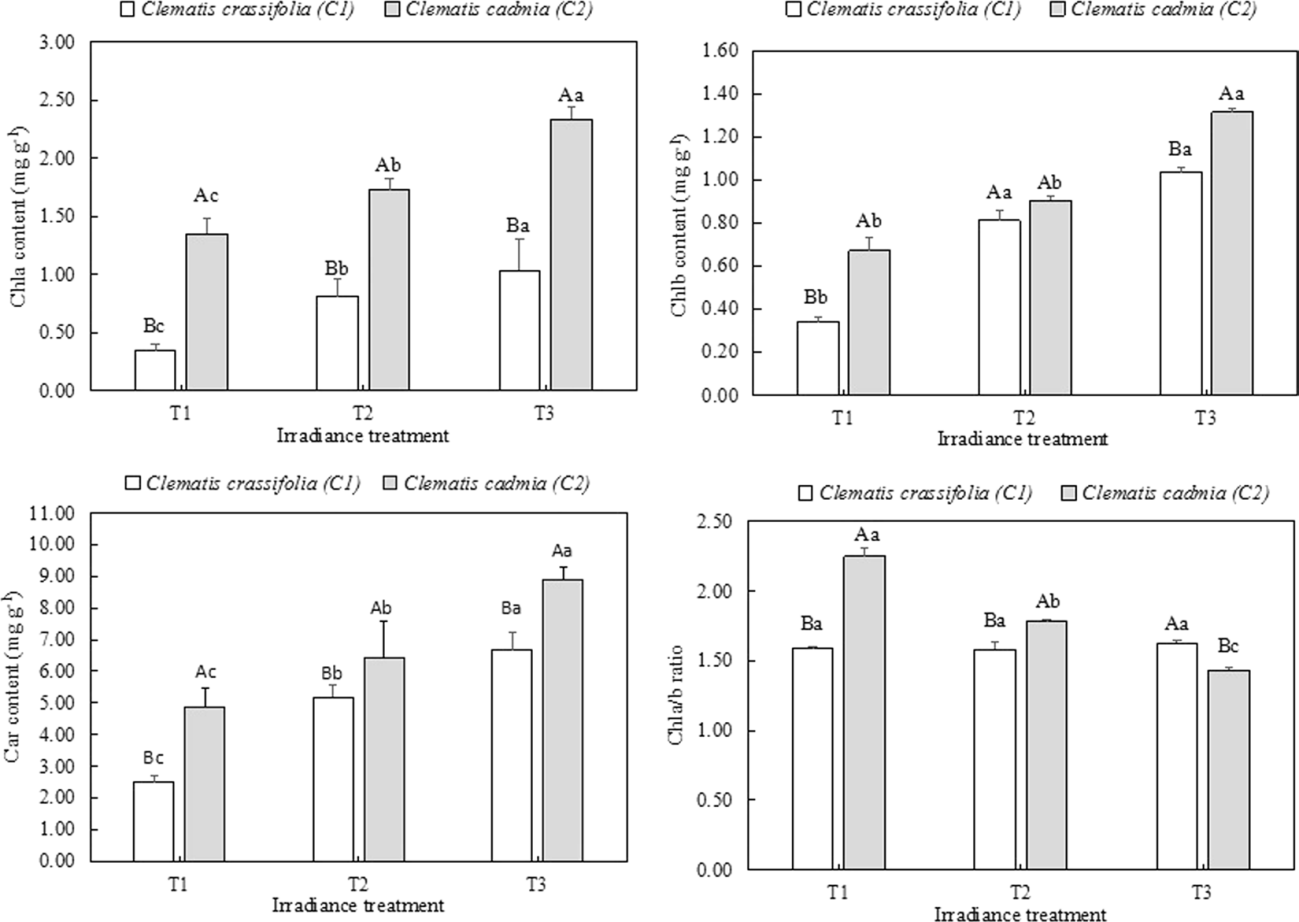
Chlorophyll *a* (chl*a*), chlorophyll *b* (chl*b*), carotenoid (car) and chlorophyll a/b ratio in *C. cadmia* and *C. crassifolia* leaves (means ± SD) grown under three different irradiances including natural irradiance (T1), moderate shade (T2); and heavy shade (T3). Different uppercase letters indicate significant difference between different *Clematis* species under same irradiance treatment at 0.05 levels; lowercase letters indicate significant difference within the same *Clematis* species under different irradiance treatments at 0.05. The values presented are the means ± SE.

Irradiance had a notable influence on the photosynthetic pigment content in *Clematis* leaves (Fig. 3). There were higher Chl*a*, Chl*b*, and carotenoid concentrations in both *C. cadmia* and *C. crassifolia* plants under T2 and T3 light treatments than in those grown under natural sunlight (T1). In *C. crassifolia*, the Chl*a* and Chl*b* content increased by 112.6% (p < 0.05) and 181.6% (p < 0.05) and by 138.2% (p < 0.05) and 204.4% (p < 0.05), respectively, under T2 and T3 light treatments compared with the Chl*a* and Chl*b* content in plants under natural light intensity (T1). While a similar trend appeared in the leaves of *C. cadmia* plants, all three pigments were at higher levels in *C. cadmia* than in *C. crassifolia* across all irradiance conditions. The carotenoid contents in *C. cadmia* leaves increased by 31.8% (p < 0.05) and 82.8% (p < 0.05), respectively, under T2 and T3 irradiance compared with the carotenoid contents in *C. cadmia* leaves under T1. Specifically, the Chla/b ratio significantly decreased (p < 0.05) with decreasing irradiance in *C. cadmia* leaves, while no significant differences in Chla/b ratio were observed in *C. crassifolia* plants across the different irradiance treatments.

### Photosynthetic parameters

The leaf photosynthetic parameters in *C. cadmia* and *C. crassifolia* were strikingly affected by the irradiance levels (Table 4). The *C. crassifolia* plants exposed to natural irradiance (T1) showed less leaf net photosynthesis (Pn) (p < 0.05), stomatal conductance (Gs) (p < 0.05), and intercellular CO_2_ concentration (Ci) (p < 0.05) and a lower transpiration rate (Tr) (p < 0.05) than those under T2 and T3 irradiance. Pn dramatically increased (p < 0.05) with decreasing irradiance in *C. crassifolia* plants, whereas a decrease (p < 0.05) in Pn in *C. cadmia* plants was observed when irradiance decreased from T1 to T3. Even under the same natural irradiance condition (T1), the Pn in *C. cadmia* was 3.18 times (p < 0.05) that in the *C. crassifolia* leaves. The variations in Gs, Ci and Tr were similar to that of Pn in the leaves of *C. cadmia* and *C. crassifolia*.

**Table 4:** Net photosynthetic rate (Pn), stomatal conductance (Gs), intercellular CO_2_ concentration (Ci), transpiration rate (Tr) of *C. crassifolia* (C1) *and C. Cadmia* (C2) plants subjected to three different levels of Irradiance including natural irradiance (T1), moderate shade (T2); and heavy shade (T3).

Treatment (irradiance)	Photosynthetic parameters			
	Pn (μmol m^−2^ s^−1^)	Gs (mmol m^−2^ s^−1^)	Tr (mmol m^−2^ s^−1^)	Ci (mmol mol^−1^)
T1C1	3.23 ± 0.68Bc	0.03 ± 0.005Ba	1.54 ± 0.18Bc	123.64 ± 1.74Ba
T2C1	4.66 ± 0.64Bb	0.05 ± 0.008Bb	2.02 ± 0.26Bb	132.05 ± 1.73Bb
T3C1	5.65 ± 0.76Aa	0.09 ± 0.01Bc	2.58 ± 0.28Aa	146.57 ± 1.94Bc
T1C2	10.27 ± 1.51Aa	0.18 ± 0.02Aa	3.85 ± 0.41Aa	193.68 ± 2.06Aa
T2C2	7.19 ± 0.88Ab	0.15 ± 0.018Ab	2.91 ± 0.31Ab	176.15 ± 1.81Ab
T3C2	4.10 ± 0.71Ac	0.13 ± 0.014Ac	1.72 ± 0.22Ac	168.55 ± 1.85Ac

Different uppercase letters indicate significant difference between different *Clematis* species under same irradiance treatment at 0.05 levels; lowercase letters indicate significant difference within the same *Clematis* species under different irradiance treatment at 0.05. The values presented are the means ± SE.

### Relative electrical conductivity (REC) and MDA content

**Figure 4:**
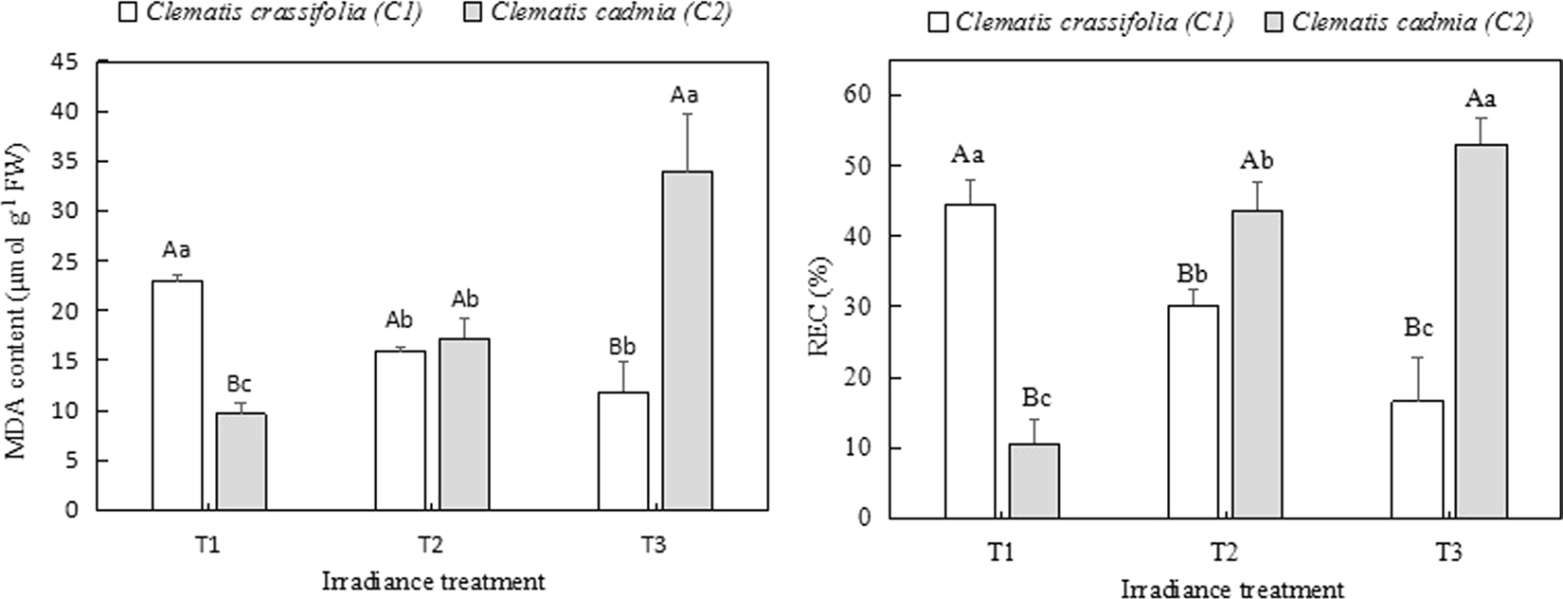
MDA content and relative electrical conductivity (REC) in *C. cadmia* and *C. crassifolia* leaves grown under different irradiance conditions. Different uppercase letters indicate significant difference between different *Clematis* species under same irradiance treatment at 0.05 levels; lowercase letters indicate significant difference within the same *Clematis* species under different irradiance treatments at 0.05. The values presented are the means ± SE.

*C. crassifolia* leaves had 32.19% (p < 0.05) and 62.83% (p < 0.05) lower REC under T2 and T3 irradiance than under natural sunlight (T1), respectively. In *C. cadmia* leaves, REC increased by 311.4% (p < 0.05) and 400.5% (p < 0.05) under T2 and T3 light intensity, respectively, compared to under natural sunlight (T1) (Fig. 4). There was 30.67% (p < 0.05) and 48.47% (p < 0.05) lower MDA content under T2 and T3 irradiance in the *C. crassifolia* leaves, respectively, than under natural irradiance (T1), while the opposite trend occurred in the *C. cadmia* leaves with the decrease in irradiance.

### H_2_O_2_ content, O_2_^−^ production rate, and antioxidant enzyme activities

**Figure 5:**
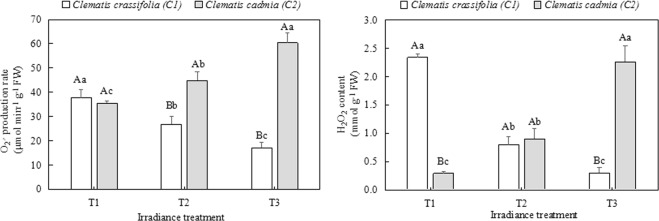
The O_2_^−^ production rate and H_2_O_2_ content in *C. cadmia* and *C. crassifolia* leaves grown under different irradiance conditions. Different uppercase letters indicate significant difference between different *Clematis* species under same irradiance treatment at 0.05 levels; lowercase letters indicate significant difference within the same *Clematis* species under different irradiance treatments at 0.05. The values presented are the means ± SE.

The H_2_O_2_ content and O_2_^−^ production rate were notably different between *C. cadmia* and *C. crassifolia* leaves (Fig. 5). The H_2_O_2_ content in *C. crassifolia* leaves decreased by 65.6% (p < 0.05) and 87.03% (p < 0.05) under T2 and T3 irradiance, respectively, compared with that under natural light intensity (T1), whereas H_2_O_2_ content increased by 210.3% (p < 0.05) and 679.3% (p < 0.05) in *C. cadmia* leaves under T2 and T3 irradiance, respectively. (Fig. 5). Similar responses were observed in the O_2_^−^ production rate.

**Figure 6:**
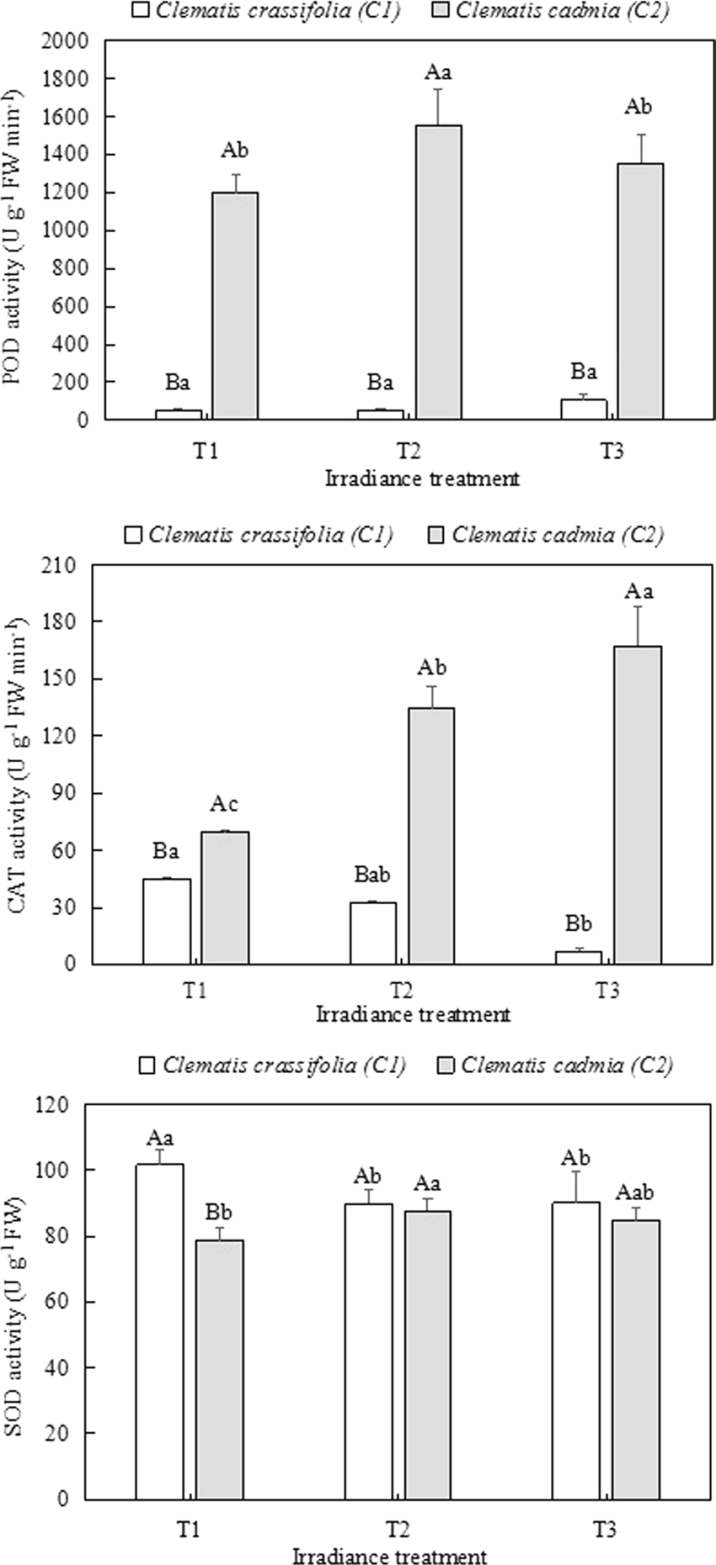
Peroxidase (POD), catalase (CAT) and superoxide dismutase (SOD) activity in *Clematis* leaves grown under three different irradiance conditions. Different uppercase letters indicate significant difference between different Clematis species under same irradiance treatment at 0.05 levels; lowercase letters indicate significant difference within the same Clematis species under different irradiance treatments at 0.05. The values presented are the means ± SE.

A marked decrease was observed in the CAT (p < 0.05) and SOD activity (p < 0.05) in *C. crassifolia* growing under T2 and T3 light treatments compared with those in the control plants growing under natural irradiance (Fig. 6). Conversely, there were 92.8% (p < 0.05) and 140.4% (p < 0.05) increases in CAT activity and 29.8% (p < 0.05) and 13.5% (p > 0.05) increases in POD activity under T2 and T3 irradiance, respectively, in *C. cadmia* compared with the activity under natural light intensity (T1). Interestingly, the SOD activity in *C. crassifolia* leaves grown under natural irradiance (T1) was higher (p < 0.05) than that in the other light treatments, and no obvious differences (p > 0.05) were found between the other irradiance treatments.

### Soluble protein content

**Figure 7:**
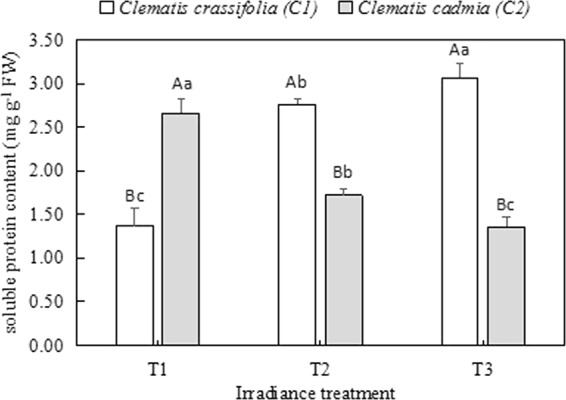
Soluble protein content (means ± SE) in *Clematis* leaves grown under three different irradiance conditions.

The soluble protein in the *C. cadmia* and *C. crassifolia* leaves differed significantly (p < 0.05) under different light intensities (Fig. 7). A significant increase (p < 0.05) was observed in the soluble protein content from the T1 to the T3 irradiance treatments in *C. crassifolia*. In contrast, there was a dramatic reduction (by 35.3% (p < 0.05) and 48.87% (p < 0.05)) in the soluble protein content under the T2 and T3 irradiance treatments, respectively, in *C. cadmia* compared with that under natural light intensity (T1).

### Relative gene expression analysis

**Figure 8:**
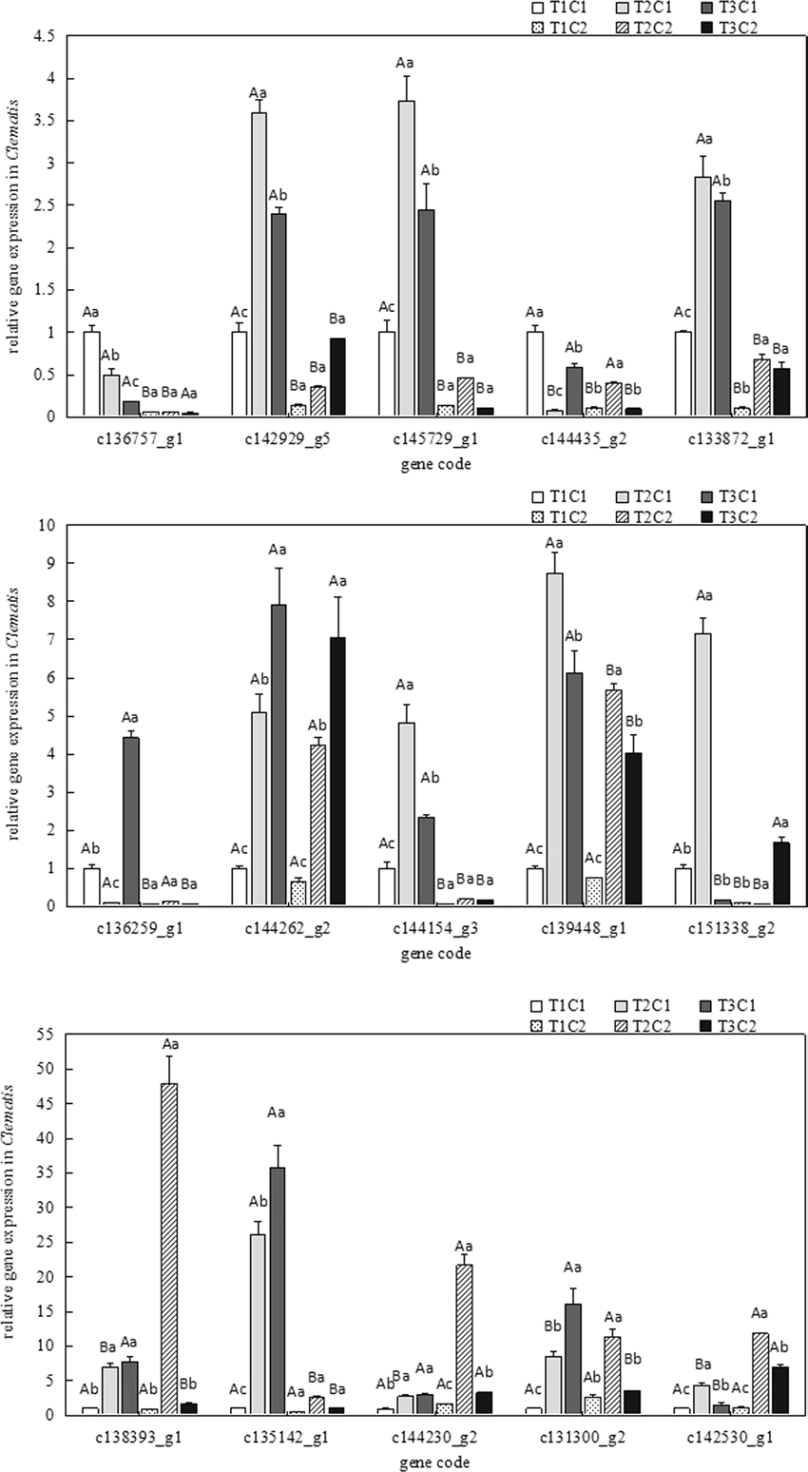
Variation in relative gene expression in *C. crassifolia* (C1) *and C. Cadmia* (C2) leaves developed under different three irradiance treatments including natural irradiance (T1), moderate shade (T2); and heavy shade (T3). The values presented are the means ± SE. Different uppercase letters indicate significant difference between different *Clematis* species under same irradiance treatment at 0.05 levels; lowercase letters indicate significant difference within the same *Clematis* species under different irradiance treatments at 0.05.

As shown in Fig. 8, the genes in *Clematis* showed differential expression in response to various irradiance levels. A total of 15 genes were analyzed for differences in gene expression in *C. cadmia* and *C. crassifolia* leaves under three irradiance levels. Three of those genes, including c136757_g1, c144230_g2 and c145729_g1, are involved in the biosynthesis of phytochrome and chlorophyll in chloroplasts. The expression of these genes was significantly different (p < 0.05) in *C. cadmia* and *C. crassifolia* under the three irradiance levels. The genes c144230_g2 and c144262_g2, which encode the receptor proteins in the Photosystem II core, were upregulated in *C. crassifolia* from T1 to T3, while the same genes were upregulated more than 5-fold (p < 0.05) in *C. cadmia* under T2 irradiance compared their levels under T1. The genes c131300_g2 and c133872_g1, which belong to the abscisic acid receptor family, were differentially upregulated in *C. crassifolia* and *C. cadmia* in response to the shaded conditions. A similar trend was observed in c133872_g1, c144154_g3 and c145729_g1 expression. The gene c139448_g1, which is associated with auxin response, first showed upregulation and then downregulation in *C. crassifolia* but showed no significant difference in *C. cadmia*. Similarly, the gene c142929_g5, which is also associated with auxin response, first showed upregulation and then downregulation in *C. crassifolia* and *C. cadmia* as the irradiance changed from T1 to T3.

## Discussion

Excessive or deficient irradiance is the most common environmental stress factor that affects a series of physiological, developmental and biochemical changes in plants, including molecular and cellular responses^37^. However, plants are also known to cope with these stress conditions by adjusting their metabolism and physiology and making specific changes in gene expression^38^. Differences in plant growth, carbon allocation, physiology and gene expression have been investigated for species suffering from different levels of irradiance stress^39^. In this study, we have illustrated the growth, physiological mechanisms and changes in some gene expression patterns of *C. cadmia* and *C. crassifolia* in response to different irradiance levels.

Irradiance that is too intense can be stressful, resulting in reduced growth, leaf fading and foliage yellowing, as shown in Fig. 2, where the *C. crassifolia* plants under natural irradiance (T1) experienced growth inhibition accompanied by lower leaf fresh weight. This is probably because higher irradiance can lead to photoinhibition and other adverse reactions in *C. crassifolia*. However, *C. cadmia* showed increased growth vigor and leaf fresh weight under higher irradiance conditions, which demonstrated that *C. crassifolia* and *C. cadmia* prefer entirely different light environments. This difference was largely caused by long-term adaptation to their contrasting ecological niches. Moreover, the specific leaf weight in the two *Clematis* species decreased with the decrease in irradiance. This is a common response in species suffering from low irradiance, indicating that plants increase light energy capture by increasing dry weight per unit leaf area; this strategy has been observed in *Torreya grandis*^25^, *Camptotheca acuminata*^40^, and wheat^41^.

Plant leaf systems possess numerous fundamental functions for adapting to different irradiances, including chlorophyll content changes and photosynthesis. The changes in pigment content in *Clematis* are shown in Fig. 3. The decreasing irradiance elevated the content of carotenoids, chlorophyll *a* and chlorophyll *b* in both species of *Clematis* but reduced the Chl*a/b* ratio in *C. cadmia*. These findings were consistent with observations in *C. acuminata*^40^, suggesting that a long exposure to high light caused pigments to degrade in *C. crassifolia*, while the content of chlorophyll *b*, as an antenna chlorophyll, increased in *C. cadmia* to absorb more light energy for photosynthesis; thus, the Chl*a/b* ratio underwent a sharp decline in *C. cadmia*^40^. Photosynthesis is correlated with chlorophyll content in plants. In our study, decreasing irradiance significantly increased the Pn, Ci, Tr, and Gs values in *C. crassifolia* but lowered those values in *C. cadmia*. These results indicated that *C. cadmia* could take full advantage of the energy under high light (T1) and suitable growth conditions, but higher light intensity (T1) would lead to an immediate interruption of growth and excessive light energy accumulation in *C. crassifolia*^42^.

Accordingly, excessive light energy could impair the photosynthetic electron transport chain and interrupt PSII electron transport. Excess ROS can be produced by the direct transfer of excitation energy from chlorophyll, causing membrane permeability dysfunction and lipid peroxidation^43^. In this study, as *C. crassifolia* and *C. cadmia* were grown under different irradiance levels for three months, we observed that high light caused higher H_2_O_2_ content and a higher O_2_^−^ production rate in *C. crassifolia*, thereby resulting in a sharp increase in MDA content and coincident increases in the relative electrical conductivity (REC); however, *C. cadmia* showed the opposite response. This phenomenon clearly suggests that an inability to adapt to light intensity could lead to either a photooxidative or an inadequate stress response in plants, closely accompanied by ROS overproduction and ultimately leading to damage to plant membranes^44^. This result was in agreement with the effects of photoinactivation and lipid peroxidation described by Takahama and Nishimura^45^. Substantial evidence has indicated that plants can alleviate and repair the damage caused by ROS through antioxidant enzymes, including SOD, POD, and CAT^40,46,47^. Our research confirmed this point in *Clematis* and showed that the POD, SOD and CAT enzyme activity increased when plants suffered from irradiance stress.

Proteins encoded by various plant genes, including most of the known enzymes (such as POD, APX and GST) and some compatible modulators of metabolic processes, perform many biological functions^48^. Our study found that light intensity significantly impacted the soluble protein content in *Clematis*; the soluble protein content increased in *C. crassifolia* grown under low irradiance but decreased in *C. cadmia* under the same conditions. Similar results were reported in microalgae^49^ and wheat^50^, which suggests that soluble protein content could represent the physiological status of plants to some extent^51^.

In general, plants respond to stresses with changes in the expression of related genes that encode proteins that provide protective effects in plants^52^. Our study focused on the responses of *C. cadmia* and *C. crassifolia* to different irradiances and screened 15 light-related genes to analyze their expression (Fig. 8). Overall, the 15 genes were differentially expressed under irradiance stress in *C. cadmia* and *C. crassifolia*. After three months of irradiance treatment, low light induced upregulated gene expression of numerous genes in *C. crassifolia*, which are involved in pigment biosynthesis, Photosystem II core protein synthesis, glycometabolism and auxin response protein synthesis. Our results in *C. crassifolia* are consistent with previous observations of some shade-tolerant plants, such as *Anoectochilus formosanus*^38^. This suggested that these genes contribute to adaptation to low light in *C. crassifolia*. PYL receptors perceive ABA intracellularly and, as a result, form ternary complexes initiating ABA signaling^53,54^. In this study, the *PYL* receptors were upregulated in *C. cadmia* grown under low light, which indicated that the *C. cadmia* plants were under stress when growing in low light (T2, T3) and that low light could activate ABA-responsive gene expression in the protoplasts, contributing to the adjustment of *C. cadmia* to the adverse environment. Interestingly, the superoxide dismutase [Cu-Zn] pathway gene was also upregulated in *C. cadmia* under low light, which indicated that *C. cadmia* increases the biosynthesis of SOD under low light. However, *C. crassifolia* showed the opposite response.

In summary, the present results confirm that the promotion of *C. crassifolia* growth under low irradiance may be related to the changes in gene expression in response to irradiance, the increased chlorophyll content and photosynthetic rate and the decreased ROS content. In addition, *C. crassifolia* and *C. cadmia* exhibit different physiological and metabolic characteristics, as well as antioxidant defense and gene expression patterns, under different irradiance conditions. This difference was largely due to the contrasting irradiance conditions in the habitats of the different species. Long-term evolution led to the differences between the species. Therefore, we proposed the hypothesis that irradiance might regulate the expression patterns of genes in these plants and might be an important environmental factor that affects the direction of plant evolution. The hypothesis will be tested in future work.
